# The influence of intergenerational support on the subjective wellbeing of the older population: the moderating effects of community environment and filial expectation

**DOI:** 10.3389/fpubh.2025.1734304

**Published:** 2026-01-12

**Authors:** Nan Zhao, Cuiying Zhu

**Affiliations:** Hunan Agricultural University, Changsha, China

**Keywords:** CFPS2020, community environment, filial piety culture, intergenerational support, subjective wellbeing, support mismatch

## Abstract

Guided by the ecological systems perspective, this study investigates how intergenerational support is associated with the subjective wellbeing (SWB) of the older population in China, emphasizing the interplay between family and community environments. Using nationally representative data from the 2020 China Family Panel Studies (CFPS), we systematically examine the effects of financial, instrumental, and emotional support on the SWB of the older population. The results indicate that self-efficacy serves as a significant mediator in this relationship, while filial expectation and community environment act as important moderating factors. Specifically, a favorable community environment can partially compensate for insufficient intergenerational support, thereby enhancing the older population wellbeing. These findings reveal the dynamic interaction between familial and community resources, extending the ecological understanding of older population wellbeing and offering empirical insights for community-based health interventions and family support policies in aging societies.

## 1 Introduction

China is undergoing a profound demographic shift characterized by rapid population aging. By 2025, the number of people aged 60 and above in China has exceeded 300 million, accounting for more than 21% of the total population, with 70% of the older population expressing concerns about their future livelihood security (1). As the proportion of the older population continues to rise, how to enhance their quality of life—particularly their subjective wellbeing (SWB)—has become a critical issue for sustainable social development. Subjective wellbeing not only directly affects the daily life experiences and social interactions of the older population but also bears on social harmony and stability.

Within China’s older population care system, the family has long been the primary source of support, with adult children playing a central role in providing financial assistance, daily care, and emotional comfort (2, 3). However, as urbanization accelerates and social structures undergo transformation, the family’s support functions have shown a gradual weakening trend. In modern society, intergenerational support no longer relies solely on face-to-face cohabitation; instead, it has taken on more diversified forms, such as remote communication, resource referral, and psychological comfort. While this shift has enriched the content of support to some extent, it has also brought about new challenges, including unequal support and emotional absence, making the relationship between intergenerational support and the older population wellbeing more complex.

As one of the key indicators of quality of life in later years, subjective wellbeing (SWB) reflects not only individuals’ overall satisfaction with life but also constitutes a core dimension of healthy aging (4). According to the WHO social determinants of health model, family intergenerational support and community environment form the two fundamental pillars influencing the health of the older population (5). The social determinants of health (SDH) refer to the socioeconomic and environmental conditions that shape individuals’ life trajectories, including income, education, social participation, housing, and community services (6). Evidence shows that, beyond family support, broader SDH such as community environments and socioeconomic resources play an important role in later-life health and wellbeing. Studies have linked socioeconomic status and community resource accessibility to multimorbidity among middle-aged and older adults, and consistently demonstrate that economic and social resources are key determinants of health (7, 8). Research also indicates that community environments significantly affect physical and mental health (9), while accessible community eldercare services can compensate for inadequate family support (10).

China’s public health system has developed a systematic mechanism for recording SDH. Under the National Basic Public Health Service Program, health records for adults aged 65 and above routinely collect information on chronic conditions, functional status, family support, lifestyle, financial strain, housing, and community resource use. These institutionalized data provide a solid empirical basis for examining how SDH shape older adults’ subjective wellbeing and justify this study’s focus on family support and community environment (11).

In the unique cultural context of filial piety in China, the older population expectations of their children (filial expectation) and the community environment may further shape the mechanism through which intergenerational support affects subjective wellbeing. On one hand, filial expectation—rooted in values and psychological anticipation—can moderate the impact of intergenerational support on wellbeing. On the other hand, a favorable community environment may serve a compensatory function when intergenerational support is insufficient, providing psychological and social resource protection for the older population.

Existing research has largely focused on single dimensions of intergenerational support and has not fully considered the moderating roles of cultural values and community factors. As a result, systematic analyses of the interaction mechanisms among intergenerational support, psychological perceptions, and community resources remain limited.

Therefore, based on data from the 2020 China Family Panel Studies (CFPS2020), this study constructs intergenerational support variables from three dimensions—financial support, daily care, and emotional comfort—to examine their impact on the subjective wellbeing of the older population. It further analyzes the mediating role of self-efficacy and the moderating effects of filial expectation and community environment. The study aims to reveal the interactive mechanisms between family and community resources in shaping older population wellbeing and to provide both theoretical and empirical evidence for the development of family- and community-based policies to enhance the wellbeing of the older population.

## 2 Literature review

Research on subjective wellbeing (SWB) has gained increasing attention as society progresses and living standards improve. People’s understanding of happiness has gradually shifted from a focus on material abundance to an emphasis on psychological fulfillment. Against this backdrop, SWB has become a core concept for measuring individuals’ life satisfaction and has been widely applied in academic research (12).

Among the older population populations in China and other countries, SWB has frequently been used in empirical studies to examine its relationship with factors such as health, mortality, family structure, and social support. For example, higher levels of SWB are significantly associated with lower all-cause mortality in the older population, and its measurement typically includes multiple subdimensions such as life satisfaction, happiness, optimism, and sense of control (13). Receiving intergenerational support—emotional, financial, or in the form of daily care—is generally linked to higher levels of SWB, with mediating variables such as perceived health playing an important role in this relationship (14).

In addition, some studies have explored differences and challenges in the measurement and operationalization of SWB. Certain research focuses on positive emotions and life satisfaction, while others incorporate both negative emotions and their balance with life satisfaction (15). Cultural differences have also been shown to influence how people define and evaluate “happiness” (16, 17). Even within a single country, differences in family environment, living arrangements, economic conditions, and social support networks can lead to significant variations in SWB among the older population (18).

Overall, the existing literature clearly indicates that SWB is a multidimensional and subjective indicator with strong predictive power for the quality of life and psychological wellbeing of the older population. However, challenges remain, including a lack of standardized measurement tools, a research focus predominantly on direct effects, and insufficient attention to cultural and social moderating factors.

The direct impact of intergenerational support on subjective wellbeing. In Chinese society, where familism is deeply rooted, filial piety is not only a moral imperative but also forms an important psychological foundation for the older population subjective wellbeing (19). Scholars generally construct intergenerational support indicators from three dimensions: financial support, daily care, and emotional comfort (20–22).

Among them, financial support can alleviate negative depressive emotions and enhance social participation among the older population (23, 24); daily care can reduce the risk of functional decline (25, 26); and emotional support helps ease feelings of loneliness and marginalization (27).

However, recent studies have also noted that the effects of intergenerational support are context-dependent and may exhibit diminishing marginal effects. First, in some urban families, “formalistic support” from children has become prevalent—although material assistance is provided, the lack of emotional companionship and high-quality interaction may lead to a psychological expectation gap among the older population (28). Second, other studies have pointed out that the impact of children’s support on wellbeing fluctuates with factors such as residential distance and intergenerational conflict (29, 30), suggesting that the linear assumption of “children’s support = wellbeing” does not always hold true.

In the field of aging research, growing evidence suggests that the community environment can moderate the impact of intergenerational support on the older population subjective wellbeing. Based on the community environment model proposed by Diez-Roux and Mair (2010), the community can be divided into two dimensions—physical environment and social environment—which influence health outcomes through pathways such as behavior and stress (31).

In the Chinese context, a study on the older population found that the community environment plays a moderating role in the relationship between informal/formal social support and mental health: when the community environment is more favorable, the positive effects of social support on mental health are stronger (i.e., the community environment buffers weak support and amplifies strong support) (32). Moreover, features of the built environment—such as community facilities, green spaces, and accessibility—have been shown to significantly moderate the relationship between family support and depressive symptoms: in areas with better community environments, family support has a stronger depressive symptom–relief effect, whereas in less favorable environments, this effect weakens (33). These empirical findings indicate that the community environment is not merely a background condition but also acts as an “enhancer” or “buffer.”

On the cultural dimension, filial expectation has also been shown to moderate the relationship between intergenerational support and the older population wellbeing. For example, a latent class analysis based on Chinese the older population revealed that when filial expectation and actual support from children are highly aligned, the older population report higher life satisfaction and lower loneliness; conversely, a gap between expectation and actual support may undermine their wellbeing. The study further found that social support (from the community or other sources) can play a compensatory role when this gap exists (34). In this research, filial expectation was discussed not only as a main effect variable but also as an interaction factor with intergenerational support, jointly shaping life satisfaction.

In summary, existing studies have examined the influencing factors of the older population wellbeing from various perspectives, but have paid insufficient attention to the underlying cultural and psychological variable of filial expectation. In particular, as community environments continue to reshape the lifestyles of the older population, there has been a lack of systematic exploration of how the wellbeing effects of intergenerational support are amplified or weakened within the value context of filial piety and specific community settings.

Building on the relationship between intergenerational support and subjective wellbeing, this study introduces two key variables—community environment and filial expectation—to uncover their moderating mechanisms. By doing so, it aims to enrich the contextual and cultural explanatory framework for understanding older population wellbeing.

## 3 Theoretical analysis and research hypothesis

The ecological systems theory posits that individual development is closely linked to the multiple layers of environmental systems in which a person is embedded, including the microsystem, mesosystem, and macrosystem (35). The microsystem refers to the most immediate living environment of the individual, such as family relationships and parent–child interactions; the mesosystem emphasizes the connections and interactions between different microsystems, such as the support networks between family and community; and the macrosystem encompasses broader sociocultural values, norms, and institutional contexts. Recent gerontological research further emphasizes that multiple layers of environmental resources jointly shape older adults’ subjective wellbeing, and that psychosocial resources interact across ecological levels (36, 37). In China, the rapid transformation of social structure and family forms has weakened the traditional intergenerational support system, increasing uncertainty in both emotional and material support for older adults (38). As a result, the community understood as a “non-therapeutic supportive environment,” increasingly functions as an alternative and compensatory resource when family-based support becomes insufficient (39).

This theory highlights that different levels of the environment not only exert independent effects on individual wellbeing but also influence psychological and behavioral states through interaction and coordination. In the Chinese social context, filial piety culture as a deeply rooted macrosystem, profoundly shapes the older population perceptions and expectations of support sources. Traditional beliefs emphasize “raising children for old-age security,” making intergenerational support the most critical resource at the microsystem level. Within contemporary urban families, the quality of intergenerational interaction remains a core micro-level mechanism shaping older adults’ emotions and wellbeing (40). Existing research also demonstrates that filial piety norms function as a cultural script structuring caregiving responsibilities, significantly influencing the psychological experience and wellbeing of older adults (41).

However, with the acceleration of urbanization and the transformation of family structures, children’s support is facing increasing emotional and spatial weakening, resulting in differentiated levels of subjective wellbeing among the older population. Meanwhile, the community, as a key component of the mesosystem, is no longer merely a direct resource affecting wellbeing. It may also moderate the impact of intergenerational support: when children’s support is insufficient, a high-quality community environment can compensate for the wellbeing gap of the older population, reflecting the interactive effects between these systems. Existing evidence shows that a supportive community environment can reduce loneliness, enhance self-efficacy, and partially offset deficits in family support (42).

**Figure 1:**
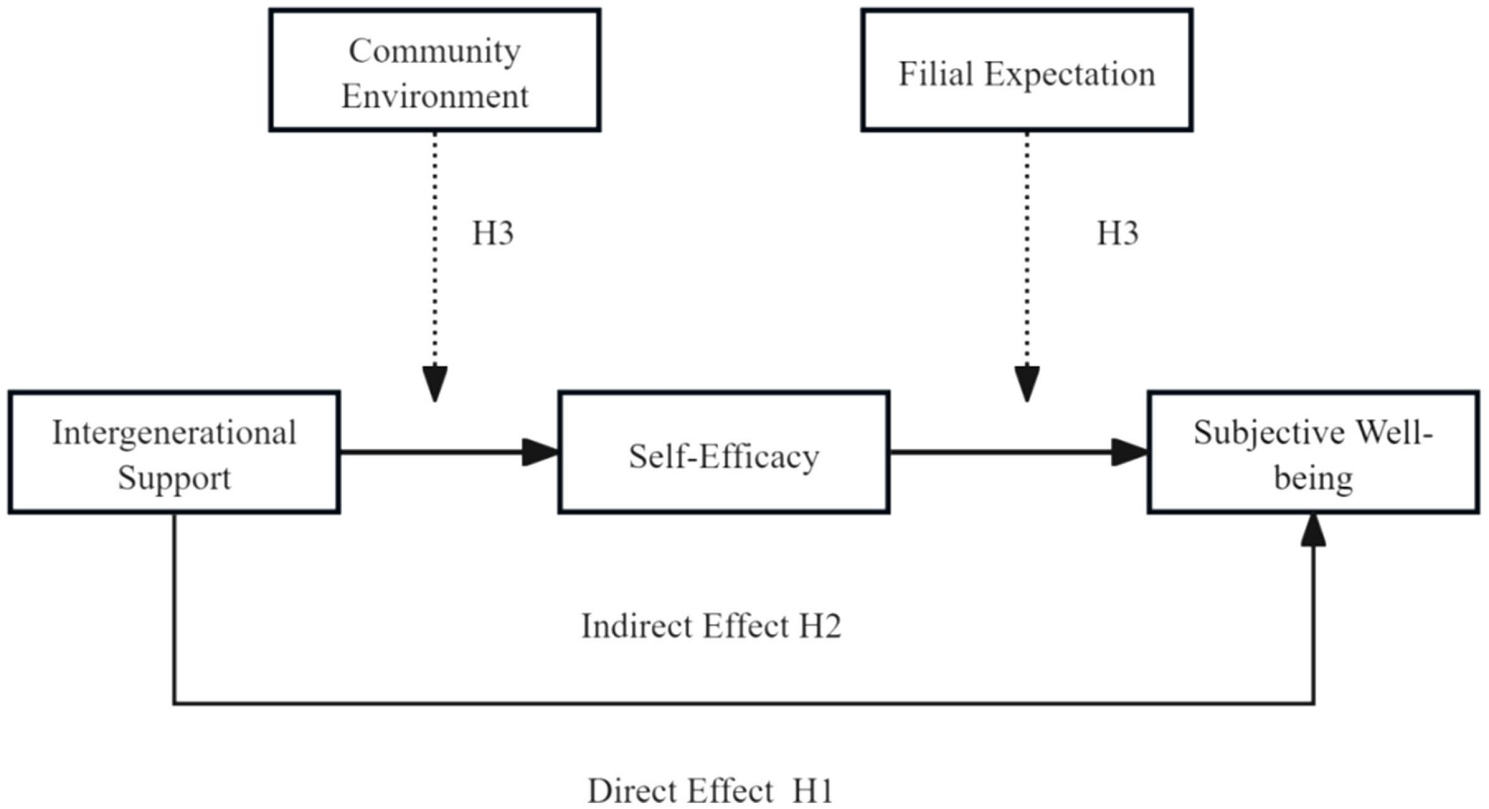
Theoretical framework.

Therefore, integrating Ecological Systems Theory with filial piety culture not only helps explain how intergenerational support and community environment influence the wellbeing of the older population at different levels, but also reveals the moderating role of community environment in the relationship between intergenerational support and wellbeing. This provides a solid theoretical foundation for understanding the multi-level formation pathways of older population wellbeing (Figure 1).

Based on these analyses, we propose the following hypotheses:

Hypothesis 1: Intergenerational support is positively associated with the subjective wellbeing of the older population. Within the microsystem, the family constitutes the most direct and immediate source of support for older individuals. High-quality intergenerational support can therefore enhance their overall wellbeing

Hypothesis 2: Intergenerational support affects the subjective wellbeing of the older population through the mediating role of self-efficacy. By improving psychological states (e.g., increasing confidence in the future), intergenerational support indirectly contributes to higher levels of wellbeing, reflecting the explanatory power of Ecological Systems Theory in understanding psychological mechanisms.

Hypothesis 3: Community environment and filial piety expectations moderate the relationship between intergenerational support and the subjective wellbeing of the older population. The meso system (community environment) and the macro system (filial piety culture) jointly shape the effects of intergenerational support: a high-quality community environment or appropriate filial piety expectations can enhance wellbeing, whereas a poor community environment or mismatched filial expectations may weaken this effect.

## 4 Method

### 4.1 Data source

This study draws on data from the 2020 China Family Panel Studies (CFPS), a nationally representative longitudinal survey administered by the Survey Research Center of Peking University. Launched in 2010 and conducted every 2 years, CFPS covers 25 provinces, autonomous regions, and municipalities across China, offering highly representative and authoritative data. The 2020 CFPS data included several modules, such as family member questionnaires, adult self-administered questionnaires, and child questionnaires, targeting individuals aged 16 and above. Information on the older population is particularly detailed, covering economic conditions, health status, psychological traits, and family interactions.

Although more recent waves of CFPS data are available, CFPS 2020 is used in this study because it uniquely includes detailed measures of community environment, which are essential for analyzing the moderating effects of community factors on the relationship between filial support and older population subjective wellbeing. Using the latest waves would omit this key variable, limiting the ability to test the proposed theoretical framework.

The study population consists of the older population aged 60 and above who have at least one living child. During sample selection, respondents without living children were excluded first. Cases with substantial missing data on key variables (e.g., subjective wellbeing, intergenerational support, and community environment) were subsequently removed. Minor missing values were addressed using multiple imputation (43). After these procedures, a total of 7,003 valid the older population respondents from across China were retained, providing a reliable reflection of the national older population population.

### 4.2 Variables description

#### 4.2.1 Dependent variable

In this study, the dependent variable is the older population subjective wellbeing (SWB). Subjective wellbeing is generally defined as an individual’s overall evaluation of their own happiness, encompassing both affective and cognitive dimensions (44, 45). Self-assessment measures are widely used due to their simplicity and their ability to directly capture personal feelings. In the present study, SWB was measured using the CFPS 2020 adult questionnaire item: “How happy do you feel about your life?” This single-item, self-reported measure is scored on a scale from 0 to 10, where 0 indicates “completely unhappy” and 10 indicates “extremely happy.” This approach provides a direct reflection of an individual’s overall sense of wellbeing and has been extensively applied in demographic, psychological, and public administration research on happiness (46, 47).

#### 4.2.2 Independent variable

The key explanatory variable in this study is intergenerational support, which refers to the multifaceted assistance that adult children provide to their parents in the form of financial, instrumental, and emotional support (48–50). Drawing on the CFPS 2020 data, intergenerational support was operationalized across three dimensions:

1. Financial support. This dimension was measured by the question: “In the past 6 months, have your children provided you with any financial assistance (including cash or in-kind support)?” Response options were coded as 1 = “Yes” and 5 = “No.” For respondents with multiple surviving children, responses were first converted to a binary variable (1 = Yes, 0 = No), and then averaged across all children. Higher values indicate a higher level of financial support.
2. Instrumental support. This was measured by the question: “In the past 6 months, have your children helped with housework or taken care of your daily needs?” The coding procedure was identical to that of financial support. The resulting average score reflects the overall level of instrumental care received from children.
3. Emotional support. This was assessed based on the question: “In the past 6 months, how often have you seen your children?” Response options ranged from 1 = “Almost every day,” 2 = “3–4 times a week,” 3 = “1–2 times a week,” 4 = “2–3 times a month,” 5 = “Once a month,” 6 = “Every few months,” to 7 = “Never.” To facilitate interpretation, this variable was reverse-coded so that higher values indicate higher contact frequency, and the average frequency across all children was used to capture the overall level of emotional support. For analysis, the frequency was categorized into three levels: (1) 1–3 times per month, (2) 4–8 times per month, and (3) more than 8 times per month.

Because CFPS 2020 collected information separately for each surviving child, variations exist in the type and intensity of support across children. Using data from only one child may introduce measurement bias. To address this, the average value across all children for each dimension was calculated to represent the total level of intergenerational support received by the older population respondent.

#### 4.2.3 Moderator variables

This study introduces filial expectation as a moderating variable to capture the deep cultural influence of filial piety on the formation of subjective wellbeing among the older population in contemporary China. Filial expectation refers to the degree to which individuals expect their children to provide support in areas such as caregiving, financial assistance, and emotional interaction. It reflects the older population internalized belief in the traditional notion of “raising children for old-age support.”

Measurement was based on relevant items from CFPS 2020, including: “to have someone to rely on in old age” (care and support), “to provide financial assistance to the family” (economic support), and “to strengthen family ties” (emotional bonding). Each item was rated on a five-point Likert scale (1 = strongly disagree, 2 = disagree, 3 = agree, 4 = strongly agree, 5 = neither agree nor disagree). Higher scores indicate stronger endorsement of filial motivations as a reason for raising children.

For analytical purposes, responses were dichotomized: participants who selected “strongly disagree” or “disagree” were categorized as having low filial expectation (coded as 0), while those who selected “agree” or “strongly agree” were categorized as having high filial expectation (coded as 1). This binary categorization allows for a clearer examination of how the relationship between intergenerational support and subjective wellbeing varies across different levels of filial expectation. In addition, using a dichotomous variable simplifies the interpretation of regression models and improves the clarity of the findings for readers.

Perceived Community Environment: The study conceptualizes “community environment” as the perceived quality of one’s living environment, measured using six items (ce1–ce6) from the 2020 wave of the China Family Panel Studies (CFPS). Consistent with prior research emphasizing subjective environmental appraisal in wellbeing studies (51), the measure covers two dimensions: (1) Perceived Physical Environment: ce1 Public facilities (e.g., perceived availability of education, healthcare, transportation); ce2 Surrounding environment (cleanliness, noise, waste conditions); ce3 Security (perceived neighborhood safety). (2) Perceived Social Environment: ce4 Neighborhood relations; ce5 Neighbor help; ce6 Attachment to community. All items were reverse coded when appropriate, standardized, and aggregated into a composite index, with higher scores indicating a better perceived community environment. The scale demonstrates excellent reliability (Cronbach’s *α* = 0.9273).

#### 4.2.4 Mediator variable

The mediating variable in this study is the self-efficacy of the older population. The concept of self-efficacy was first introduced by Canadian psychologist Albert Bandura (52). It refers to an individual’s perceived ability and confidence when facing environmental challenges. Self-efficacy reflects not only one’s evaluation of personal competence but also the belief in effectively applying these abilities to achieve goals in specific situations. A higher level of self-efficacy is typically associated with stronger psychological resilience, lower feelings of helplessness, and greater subjective wellbeing. In this study, self-efficacy is measured using the “confidence in the future” indicator from the 2020 wave of China Family Panel Studies, which captures individuals’ positive expectations and perceived personal capability. Higher scores indicate stronger perceived self-efficacy among the older population.

#### 4.2.5 Control variables

The subjective wellbeing of the older population is shaped by a combination of individual characteristics, family factors, and social environmental conditions. To accurately identify the effect of intergenerational support on the subjective wellbeing of the older population, this study includes several demographic and socioeconomic characteristics as control variables. These variables cover gender, age, education, marital status, health status, place of residence, economic situation, number of children, and pension security.

##### 4.2.5.1 Gender

Gender, as a fundamental social attribute, is closely linked to the distribution of social roles, access to resources, and psychological experiences. Gender differences may lead to variations in individuals’ social support networks, economic status, and emotional expression, which in turn shape the formation and perception of subjective wellbeing. In this study, gender is operationalized as a binary variable, with females coded as 0 and males coded as 1, to examine potential differences in subjective wellbeing between different gender groups.

##### 4.2.5.2 Age

Age not only reflects an individual’s demographic characteristics but is also closely associated with their life course, social experiences, and health status. As people grow older, changes may occur in physical functioning, social roles, and psychological adaptation, all of which can influence subjective wellbeing. In this study, age is constructed as a continuous variable based on respondents’ reported age. Following the definition of the older population population by the World Health Organization, the sample is restricted to individuals aged 60 and above to highlight the characteristics of subjective wellbeing in later life.

##### 4.2.5.3 Educations

Educational attainment is an important indicator of an individual’s human capital and their ability to access social resources. Individuals with higher levels of education typically have broader access to information and stronger adaptive capacities, which enable them to better cope with life pressures and social changes in later life, thereby positively influencing their subjective wellbeing. In this study, the highest level of education reported by respondents is used to measure educational attainment. It is then recoded into a binary variable: illiterate (including illiterate and semi-illiterate) is coded as 0, and literate (having received formal or informal basic education) is coded as 1. This approach provides a clear basis for comparing differences in basic educational levels among the older population and facilitates the analysis of how education affects subjective wellbeing.

##### 4.2.5.4 Marriage

Marriage is not only a social institution but also an important source of emotional connection and social support. Married individuals often enjoy more stable emotional companionship and economic support, which can enhance their subjective wellbeing. In contrast, those who are unmarried, divorced, or widowed may experience higher levels of loneliness and emotional deprivation. In this study, marital status is recoded as a binary variable: not married (including unmarried, divorced, or widowed) is coded as 0, and married is coded as 1.

##### 4.2.5.5 Health

Health status is one of the key factors affecting the quality of life and psychological wellbeing of the older population. Good physical health not only enhances individuals’ ability to manage daily activities independently but also increases their willingness to participate in social life, thereby improving their subjective wellbeing. In this study, health status is measured based on respondents’ self-rated health and categorized into three levels: 0 = unhealthy, 1 = fair, and 2 = healthy.

##### 4.2.5.6 Residence

The urban–rural divide is often closely related to differences in infrastructure, public service provision, and the distribution of social resources. The older population living in urban areas typically have access to more comprehensive community services and better healthcare coverage, while those in rural areas may rely more heavily on family support. In this study, place of residence is coded as a binary variable, with rural areas coded as 0 and urban areas coded as 1, to control for the potential impact of urban–rural structural differences on subjective wellbeing.

##### 4.2.5.7 Incomes

Economic resources are a fundamental basis for ensuring the quality of life and enhancing wellbeing in later life. Income level not only affects the material living conditions of the older population but also shapes their sense of financial security and perceived social status. In this study, a subjective income level variable is constructed based on respondents’ answers to the question, “How would you rate your income level compared to others in your area?” The scale ranges from 1 (very low) to 5 (very high). Compared with objective income measures, subjective income evaluation better reflects individuals’ economic perceptions and their experienced wellbeing.

##### 4.2.5.8 Number of children

The number of children is closely related to family structure and affects the availability of resources for emotional support, financial assistance, and daily care in later life. In general, having more children may provide multiple sources of intergenerational support, but it can also dilute the intensity of support from each individual child. In this study, the number of surviving children reported by respondents is included as a continuous control variable.

##### 4.2.5.9 Pension security

The level of pension security is an important indicator of how well social welfare systems support the older population. Individuals with comprehensive pension coverage tend to have greater financial stability and a stronger sense of security, which can enhance their subjective wellbeing. In this study, pension security is measured by whether respondents are enrolled in a basic pension insurance program, with “0” indicating no coverage and “1” indicating coverage. The CFPS items used to construct the study variables are summarized in Table 1.

**Table 1:** Descriptive statistics of key variables.

Variables	Items	Definition	Coding	α
SWB	qm2016	How happy do you feel	0–10, completely unhappy = 0, extremely happy = 10	–
Intergenerational support				–
Financial support	qf301	In the past 6 months, children provided you with financial help	Yes = 1, no = 0	–
Instrumental support	qf303	In the past six months, children provided daily care	Yes = 1, no = 0	–
Emotional support	qf305	In the past 6 months, how often have you seen your children	1 = 1–3 times per month, 2 = 4–8 times per month, 3 = more than 8 times per month.	-
Community environment	ce1–ce6	ce1. Public facilities; ce2. Surrounding environment; ce3. Security; ce4. Neighborhood relations; ce5. Neighbor help; ce6. Attachment to community	1–5, very good = 1, very bad = 5, all items were reverse coded	0.9273
Filial expectation	qme201 qme203 qme209	qme201. Have children so that someone helps in old age; qme203. Have children to provide financial support; qme209. Have children to increase kinship ties	Agree or strongly agree = 1, strongly disagree or disagree = 0	0.9943
Self-efficacy	qn12016	Confidence in future	1-5, very little confidence = 1, a lot of confidence = 5	–

Community environment *α* = 0.9287 and filial expectation *α* = 0.9943 show high internal consistency. Other variables are single-item measures and thus not applicable for α.

### 4.3 Model construction

#### 4.3.1 Ordinary least squares

This study employs an Ordinary Least Squares (OLS) regression model, following the approach of Ferrer and Frijters (53) and related research on older population wellbeing, to examine the impact of intergenerational support on the subjective wellbeing of the older population. The regression model is specified as follows:


$${\text{Happiness}}_{i}=\alpha +\beta 1S_{i}+\gamma^{'}X_{i}+\varepsilon_{i}$$ (1)

Where Happiness_i_ represents the subjective wellbeing of older population individual i; Si denotes the total intergenerational support score, including financial, instrumental, and emotional support; β_1_ is the key coefficient of interest, capturing the marginal effect of intergenerational support on older population wellbeing; Xi is a vector of control variables, including age, gender, marital status, household registration, education level, personal income, health status, family size, and other relevant factors; *α* is the constant term; and ε_i_ is the random error term. This model corresponds to research hypothesis H1.

#### 4.3.2 Mediation effect model

To further explore the underlying psychological mechanisms, this study introduces self-efficacy as a mediating variable and constructs a mediation effect model to test H2. The specific model is as follows:


$$M_{i}=a_{0}+a_{1}S_{i}+a^{'}X_{i}+v_{i}$$ (2)

$${\text{Happiness}}_{i}=c_{0}+c_{1}S_{i}+b_{1}M_{i}+c^{'}X_{i}+\varepsilon_{i}$$ (3)

Where M_i_ represents the self-efficacy level of the older population i; a_1_ denotes the effect of intergenerational support on self-efficacy; b_1_ indicates the effect of self-efficacy on subjective wellbeing; and c_1_ represents the direct effect of intergenerational support on subjective wellbeing. If both a_1_ and b_1_ are statistically significant, this suggests that self-efficacy plays a significant mediating role in the relationship between intergenerational support and subjective wellbeing. ν_i_ denotes the random error term, and the meanings of the other symbols remain the same as previously defined.

#### 4.3.3 Moderation effect model

Considering the moderating effects of community environment (mesosystem) and filial expectations (macrosystem) on the main effect, interaction terms are further introduced to construct the following dual moderation model:


$$\begin{array}{l} {\text{Happiness}}_{i}=\alpha +\beta_{1}S_{i}+\beta_{2}C_{i}+\beta_{4}F_{i}+\beta_{3}(S_{i}\times C_{i})\\ +\beta_{5}(S_{i}\times F_{i})+\beta_{6}(C_{i}\times F_{i})+\beta_{7}(S_{i}\times C_{i}\times F_{i})\\ +\gamma^{'}X_{i}+\varepsilon_{i} \end{array}$$ (4)

In this model, C_i_ represents the quality of the community environment in which the older population resides, including the availability of public facilities, neighborhood relations, and the overall community atmosphere. S_i_ × C_i_ denotes the interaction term between intergenerational support and community environment, and β_3_ is the moderation coefficient indicating the direction and strength of the moderating effect of community environment on the relationship between intergenerational support and subjective wellbeing. F_i_ represents the level of filial expectations, which reflects the older population cultural expectations regarding children’s support and emotional reciprocity. β_5_ is the moderation coefficient capturing the amplifying or attenuating role of filial expectations in the association between intergenerational support and wellbeing. C_i_ × F_i_ denotes the interaction between community environment and filial expectations, while S_i_ × C_i_ × Fi times represents the three-way interaction term, which captures the joint moderating effect of filial expectations and community environment on the impact of intergenerational support. β_7_ is the key moderation coefficient. The other symbols are defined as above.

## 5 Results

### 5.1 Summary statistics

To ensure the scientific validity of variable selection and the reliability of the data, this study conducted descriptive statistical analyses of all key variables. Table 2 presents the means, standard deviations, minimum values, and maximum values for the independent variables, dependent variable, interaction terms, and major control variables.

**Table 2:** Summary statistics.

Variables	Obs	Mean	Std.dev.	Min	Max
SWB	7,003	7.725	2.146	0.000	10.000
Intergenerational support	7,003	0.249	0.278	0.001	1.000
Financial support	7,003	0.453	0.443	0.000	2.000
Instrumental support	7,003	0.298	0.393	0.000	1.000
Emotional support	7,003	3.100	1.942	0.000	6.000
Community environment	7,003	0.346	0.363	0.002	1.000
Filial expectation	7,003	0.250	0.274	0.002	1.000
Self-efficacy	7,003	4.173	0.967	1.000	5.000
Gender	7,003	0.544	0.498	0.000	1.000
Age	7,003	67.914	5.740	60.000	95.000
Educations	7,003	5.692	4.632	0.000	19.000
Marriage	7,003	0.821	0.383	0.000	1.000
Health	7,003	3.346	1.259	1.000	5.000
Residence	7,003	0.501	0.500	0.000	1.000
Incomes	7,003	5.204	12.244	1.000	79.000
Number of children	7,003	2.082	1.178	0.000	8.000
Pension security	7,003	0.228	0.420	0.000	1.000

### 5.2 Baseline regression

We employed an ordinary least squares (OLS) regression model to examine the association between intergenerational support and subjective wellbeing among the older population, and further explored the specific effects of different types of intergenerational support. The regression results are presented in Table 3.

**Table 3:** The effect of intergenerational support on SWB of the older population.

Variables	(1)	(2)	(3)	(4)	(5)	(6)
Intergenerational support	0.108	1.646***				
	(0.092)	(0.297)				
Financial support			0.374***			0.310***
			(0.057)			(0.058)
Instrumental support				0.383***		0.166**
				(0.063)		(0.069)
Emotional support					0.099***	0.081***
					(0.013)	(0.014)
Gender		0.683***	−0.165***	−0.175***	−0.155***	−0.148***
		(0.164)	(0.053)	(0.053)	(0.053)	(0.053)
Age		0.033***	0.034***	0.033***	0.033***	0.034***
		(0.005)	(0.005)	(0.005)	(0.005)	(0.005)
Educations		0.022***	0.017***	0.019***	0.014**	0.016***
		(0.006)	(0.006)	(0.006)	(0.006)	(0.006)
Marriage		0.402***	0.413***	0.444***	0.398***	0.409***
		(0.068)	(0.068)	(0.068)	(0.068)	(0.068)
Health		−0.384***	−0.389***	−0.380***	−0.373***	−0.377***
		(0.020)	(0.020)	(0.020)	(0.020)	(0.020)
Residence		0.089*	0.079	0.065	−0.025	−0.002
		(0.052)	(0.052)	(0.052)	(0.054)	(0.054)
Incomes		0.007***	0.007***	0.008***	0.008***	0.008***
		(0.002)	(0.002)	(0.002)	(0.002)	(0.002)
Number of children		0.011	−0.007	0.012	0.013	−0.006
		(0.022)	(0.022)	(0.022)	(0.022)	(0.022)
Pension security		−0.227***	−0.227***	−0.231***	−0.232***	−0.222***
		(0.060)	(0.059)	(0.059)	(0.059)	(0.059)
Constant	7.698***	5.473***	6.210***	6.195***	6.099***	5.899***
	(0.034)	(0.380)	(0.342)	(0.342)	(0.342)	(0.343)
Observations	7,003	7,003	7,003	7,003	7,003	7,003
*R*-squared	0.000	0.070	0.072	0.071	0.073	0.079

Standard errors in parentheses, ***p < 0.01, **p < 0.05, *p < 0.1.

In Column (1), when only the overall intergenerational support variable was included without any covariates, the coefficient was 0.108, indicating a positive association between intergenerational support and subjective wellbeing, although the effect was not statistically significant. In Column (2), after adding control variables, the coefficient of intergenerational support increased markedly to 1.646 and reached the 1% significance level, suggesting that intergenerational support has a robust positive effect on subjective wellbeing once individual characteristics are controlled for. This finding highlights the important role of children’s support in enhancing life satisfaction and wellbeing among the older population.

Columns (3) through (6) further examine the effects of different types of intergenerational support—namely financial support, instrumental care, and face-to-face contact. In the baseline models without control variables, all three types of support were significantly and positively associated with subjective wellbeing. Financial support showed the largest coefficient (0.374, *p* < 0.01), followed by instrumental care (0.383, *p* < 0.01) and face-to-face contact (0.099, *p* < 0.01). After controlling for gender, age, education level, marital status, health status, urban–rural residence, subjective economic status, number of children, and pension coverage, the coefficients remained significant but decreased slightly: 0.310 (*p* < 0.01) for financial support, 0.166 (*p* < 0.05) for instrumental care, and 0.081 (*p* < 0.01) for face-to-face contact.

These results indicate that intergenerational support exerts a stable and significant positive effect on the older population subjective wellbeing, even after accounting for a range of demographic and socioeconomic characteristics.

Specifically, among the three types of intergenerational support, financial support exerts the strongest positive effect on the older population subjective wellbeing. This may be because financial assistance is associated with reduced living pressures, greater feelings of security, and increases life satisfaction. The effect of instrumental care decreases slightly after controlling for covariates but remains statistically significant, indicating that children’s provision of daily care and assistance substantially is positively linked to the older population wellbeing. The coefficient for face-to-face contact also declines marginally yet remains significant, suggesting that emotional connection and interaction show a stable and positive association with psychological wellbeing in later life.

The effects of control variables align with expectations: the older population who are male, married, better educated, economically better off, and in good health report higher levels of subjective wellbeing. In contrast, poor health and participation in pension schemes (which may reflect weaker physical conditions or higher economic dependence) are associated with lower levels of subjective wellbeing. These findings further confirm the importance of individual characteristics in shaping wellbeing.

Overall, the regression results support our hypotheses: intergenerational support—particularly financial support, instrumental care, and emotional interaction—has a significant and positive impact on the older population subjective wellbeing. This result is consistent with previous research. For example, Peng et al. (54)found that different types of intergenerational support are significantly associated with life satisfaction among the older population. Similarly, studies in Southeast Asia have shown that financial assistance, caregiving, and emotional support significantly improve subjective wellbeing in later life (55, 56) (Table 4).

**Table 4:** Robustness tests.

Variables	(1)	(2)	(3)	(4)
	Changed dependent variable		Changed regression model	
Intergenerational support	0.016	0.915***	0.080*	0.830***
	(0.049)	(0.164)	(0.047)	(0.159)
Contro variables	No	Yes	No	Yes
/cut1	−2.398***	−1.556***	−2.031***	−1.038***
	(0.050)	(0.215)	(0.036)	(0.204)
/cut2	−1.961***	−1.104***	−1.557***	−0.548***
	(0.034)	(0.212)	(0.027)	(0.202)
/cut3	−0.868***	0.029	−0.580***	0.472**
	(0.021)	(0.211)	(0.020)	(0.202)
/cut4	0.002	0.936***	0.295***	1.386***
	(0.019)	(0.211)	(0.019)	(0.202)
Observations	7,003	7,003	7,003	7,003
*R*2	0.002	0.028	0.002	0.028

Standard errors in parentheses, ***p < 0.01, **p < 0.05, *p < 0.1.

### 5.3 Robustness tests

To ensure the robustness of the results, two approaches were employed. First, the dependent variable was replaced: life satisfaction was used instead of subjective wellbeing (SWB). Based on the survey question “How satisfied are you with your life?,” responses were rated on a five-point scale, where “1” represents “very dissatisfied” and “5” represents “very satisfied.” The results show that, without control variables, the coefficient of intergenerational support is 0.016 and not statistically significant. After including control variables, the coefficient increases to 0.915 and is significant at the 1% level. This indicates that even when the measurement of subjective wellbeing is changed, the positive effect of intergenerational support on life satisfaction remains robust. It further suggests that support from children plays a significant role in enhancing the life satisfaction of the older population.

Second, the model was changed. Since subjective wellbeing is scored on a scale of 0–10, we recoded the variable as follows: 0–2 as “very unhappy,” 3–4 as “fairly unhappy,” 5–6 as “neutral,” 7–8 as “fairly happy,” and 9–10 as “very happy,” and applied an ordered probit (oprobit) model. The results show that, without control variables, the coefficient of intergenerational support is 0.080 and significant at the 10% level; after including control variables, the coefficient increases to 0.830 and is significant at the 1% level. This further confirms the robustness of the positive relationship between intergenerational support and the older population self-rated wellbeing.

In addition, the cut values (cut1–cut4) exhibit a reasonable and consistent trend: after adding control variables, all cut values increase significantly. This indicates that as subjective wellbeing or self-rated health improves, the distribution of the older population wellbeing shifts structurally, which is consistent with the direction of the main effect.

### 5.4 Heterogeneity analysis

To further test the robustness of the baseline regression results and reveal group differences in the effect of intergenerational support on the older population subjective wellbeing, this study conducts a heterogeneity analysis based on two basic demographic characteristics: gender and place of residence. The results are presented in Table 5.

**Table 5:** Heterogeneity analysis by gender and residence.

Variables	(1)	(2)	(3)	(4)
	Female	Male	Rural	Urban
	SWB	SWB	SWB	SWB
Intergenerational support	1.833***	4.806**	2.172***	1.507***
	(0.312)	(1.020)	(0.485)	(0.375)
Constant	5.778***	5.564***	6.026***	4.846***
	(0.559)	(0.478)	(0.584)	(0.495)
Observations	3,196	3,807	3,493	3,510
*R*-squared	0.086	0.061	0.062	0.083

Standard errors in parentheses; ***p < 0.01, **p < 0.05, *p < 0.1.

First, the heterogeneity analysis by gender shows that intergenerational support has a significant positive effect on the subjective wellbeing of older women, with a coefficient of 1.833, significant at the 1% level. For men, the coefficient is 4.806 and significant at the 5% level. This indicates that while intergenerational support positively associated with subjective wellbeing for both male and female the older population, the effect is more pronounced among men. This may be related to gender differences in family roles and emotional needs: older women often play a role in maintaining emotional ties within the family and rely more on emotional support, whereas older men depend more on economic and caregiving support. As a result, receiving intergenerational support leads to a greater increase in their subjective wellbeing.

Second, the heterogeneity analysis by place of residence shows that intergenerational support has a significant positive effect on the subjective wellbeing of the older population in both urban and rural areas. In rural areas, the regression coefficient of intergenerational support is 2.172 and significant at the 1% level; in urban areas, the coefficient is 1.507, also significant at the 1% level.

Notably, the effect of intergenerational support is stronger for rural the older population than for their urban counterparts. This may be due to the relative scarcity of public service resources and the limited social support networks in rural areas, making children the primary source of both emotional and material support for the older population. In contrast, urban the older population have access to more external support and opportunities for social participation. While children’s support remains important, its marginal effect is relatively smaller.

In summary, the heterogeneity analysis further confirms both the robustness and the differentiated effects of intergenerational support on the subjective wellbeing of the older population. Gender and urban–rural background play important moderating roles in this relationship: male and rural the older population rely more heavily on intergenerational support, leading to a greater increase in their wellbeing, while female and urban the older population exhibit stronger emotional attachment and have more diverse support sources. This finding provides important empirical evidence for formulating differentiated social and psychological service policies for various older population groups.

### 5.5 Mechanism analysis

The regression results are presented in Table 6, with columns (1) to (6) showing the effects of intergenerational support, self-efficacy, community environment, and filial expectation on the subjective wellbeing of the older population, as well as the relevant interaction effects. Intergenerational support exhibits a significant positive effect on both filial expectation and self-efficacy. In column (1), the coefficient of intergenerational support on filial expectation is 0.874 and is significant at the 0.1% level, indicating that the more substantial the support provided by children, the higher the older population expectations regarding filial duty. Column (3) further shows that the coefficient of intergenerational support on self-efficacy is 1.032, also significant at the 0.1% level, suggesting that adequate intergenerational support is associated with greater confidence in coping with life pressures and challenges, thereby increasing their self-efficacy.

**Table 6:** Regression results for mediating and moderating effects.

Variables	(1)	(2)	(3)	(4)	(5)	(6)
	Filial expectation	SWB	Self-efficacy	SWB	SWB	SWB
Intergenerational support	0.874***	−0.956	1.032***	0.785***	1.600***	4.193***
	(0.003)	(1.222)	(0.135)	(0.276)	(0.295)	(0.673)
Filial expectation		2.979**				
		(1.357)				
Self-efficacy				0.834***		
				(0.024)		
Community environment					1.802***	3.911***
					(0.214)	(0.537)
IS × CE						7.156***
						(1.671)
Constant	0.064***	5.284***	3.798***	2.307***	4.258***	2.097***
	(0.003)	(0.389)	(0.172)	(0.364)	(0.404)	(0.646)
Observations	7,003	7,003	7,003	7,003	7,003	7,003
*R*-squared	0.996	0.071	0.056	0.203	0.079	0.082

Standard errors in parentheses; ***p < 0.01, **p < 0.05, *p < 0.1.

#### 5.5.1 Mediation effect analysis

Self-efficacy has a significant positive effect on subjective wellbeing. As shown in column (4), the coefficient of self-efficacy is 0.834, significant at the 0.1% level. This indicates that the older population with higher self-efficacy are more likely to perceive meaning and satisfaction in life, thereby experiencing greater subjective wellbeing. These findings are consistent with Albert Bandura’s self-efficacy theory, which posits that self-efficacy is associated with individuals’ sense of control and psychological resilience, ultimately contributing to higher levels of wellbeing.

#### 5.5.2 Moderating effect analysis

The community environment plays a crucial role in enhancing subjective wellbeing. As shown in columns (5) and (6), the coefficients for the social environment are 1.802 and 3.911, respectively, both significant at the 0.1% level. This indicates that a favorable community environment—such as well-developed public facilities, harmonious interpersonal relationships, and a strong sense of security—can directly enhance the older population subjective wellbeing. Meanwhile, the interaction term between intergenerational support and social environment has a coefficient of 7.156 and is highly significant, suggesting a substantial moderating effect: the positive impact of intergenerational support on wellbeing is amplified in better community environments. Filial expectations also exert a significant positive effect on subjective wellbeing. In column (2), the coefficient of filial expectation is 2.979, significant at the 5% level. This indicates that the value of “intergenerational reciprocity” embedded in traditional filial culture remains an important psychological foundation for the older population wellbeing. When the older population expectations of filial behavior are met, they are more likely to experience emotional satisfaction and a sense of personal value (Table 6).

## 6 Discussion

### 6.1 Direct effect of intergenerational support on older population subjective wellbeing

The empirical results indicate that intergenerational support has a significant positive effect on the subjective wellbeing of the older population (*β* = 0.326, *p* < 0.001). This suggests that greater financial assistance, caregiving, and emotional companionship from children are associated with higher levels of life satisfaction and wellbeing among the older population. This finding is highly consistent with previous studies (3, 22), further confirming that in a family-centered sociocultural context, intergenerational relationships remain a core determinant of the older population wellbeing.

In traditional Chinese society, the culture of filial piety emphasizes children’s responsibility to provide care and support for their parents, serving as a fundamental pillar for maintaining family stability and ensuring the quality of life of the older population. Unlike Western societies, which place greater emphasis on individual independence and self-fulfillment, the wellbeing of Chinese older population is more deeply rooted in family interactions and emotional bonds. Particularly in the context of persistent urban–rural disparities in social security, high-quality intergenerational support remains a crucial resource for sustaining both the living standards and emotional wellbeing of many the older population.

### 6.2 Mediating effect of self-efficacy

Further mediation analysis reveals that intergenerational support significantly associated with higher self-efficacy (*β* = 0.284, *p* < 0.001), and self-efficacy, in turn, has a significant positive effect on subjective wellbeing (*β* = 0.275, p < 0.001). The indirect effect is 0.078, accounting for 23.9% of the total effect, indicating that self-efficacy plays a partial mediating role in the relationship between intergenerational support and the older population subjective wellbeing.

This finding reveals the psychological mechanism through which intergenerational support is associated with wellbeing. According to self-efficacy theory, positive external support can enhance individuals’ sense of control and confidence in life. When the older population receive stable and sustained support from their children, they are more likely to respond proactively to uncertainties and challenges, thereby strengthening their psychological resilience and emotional stability, which in turn improves their wellbeing. This conclusion is consistent with the findings of Li & He (14) and further suggests that material or emotional support alone cannot fully explain the formation of wellbeing—psychological perception processes are a critical component.

### 6.3 Moderating effects of community environment and filial expectation

In the moderation analysis, both community environment and filial expectations significantly moderated the relationship between intergenerational support and subjective wellbeing. Specifically, the interaction between community environment and intergenerational support was significant (*β* = 0.116, *p* < 0.01), and the interaction between filial expectations and intergenerational support was also significant (β = 0.094, *p* < 0.05).

Further interaction effect plots reveal that the positive impact of intergenerational support on subjective wellbeing is more pronounced when the community environment is favorable, whereas this effect weakens considerably in less supportive community contexts. This suggests that meso-level community conditions can amplify or dampen the effects of family support. In addition to the statistical moderation effect, a favorable community environment may compensate for insufficient family support through several theoretical mechanisms. First, community services such as health management, recreational activities, and daily-life assistance can function as alternative resource provision, filling the gaps left by limited financial or caregiving support from children (57). Second, community organizations and social activities promote enhanced social integration, helping the older population build social ties, reduce loneliness, and obtain emotional support that would otherwise come primarily from family members (58). Third, accessible facilities, safety infrastructure, and supportive neighborhood networks strengthen the older population sense of security, autonomy, and efficacy, thereby mitigating the negative consequences of inadequate family support (59). These mechanisms suggest that community environments do not merely amplify family support but can also provide partial functional or emotional substitutes when family resources are insufficient.

Moreover, among individuals with higher levels of filial expectations, the relationship between intergenerational support and subjective wellbeing is significantly stronger, while it is relatively weaker among those with lower filial expectations. This indicates that filial piety, as a macro-level cultural value, plays an important moderating role by shaping psychological expectations and value orientations of the older population.

This finding highlights the contextual and differential nature of the impact of intergenerational support. A favorable community environment not only provides public facilities and opportunities for social interaction but also helps alleviate family caregiving burdens, thereby amplifying the positive effects of intergenerational support. At the same time, a higher level of filial expectations reflects the older population strong endorsement of their children’s filial obligations, leading to greater psychological satisfaction when such support is received.

In summary, this study reveals that intergenerational support, self-efficacy, community environment, and filial expectations together form a multi-level mechanism influencing the subjective wellbeing of the older population.

At the micro level, intergenerational support serves as a direct source of wellbeing, providing emotional and material foundations for family life and quality of living.

At the psychological level, self-efficacy plays a significant mediating role between intergenerational support and wellbeing, underscoring the bridging function of subjective psychological perceptions in the formation of happiness.

At the meso and macro levels, the moderating roles of community environment and filial expectations indicate that the effectiveness of family support is shaped by broader social and cultural contexts.

These findings are consistent with the core tenets of ecological systems theory, which emphasize that individual wellbeing is shaped by the interactions among micro-, meso-, and macro-level systems. Relying solely on family caregiving is no longer sufficient to meet the needs of an aging society. Instead, a “family–community–culture” integrated support system is essential to generate synergistic effects. This approach not only contributes to improving the quality of life for the older population but also provides solid empirical evidence and policy implications for improving a diversified support system in the context of population aging in China.

## 7 Conclusions, policy implications, and limitations

### 7.1 Conclusion

In the context of a rapidly aging society, enhancing the subjective wellbeing of the older population has become a crucial issue in social policy and public governance. Drawing on data from the 2020 China Family Panel Studies (CFPS), this study systematically examines the mechanisms through which intergenerational support, self-efficacy, community environment, and filial expectations influence the subjective wellbeing of the older population. The findings show a significant positive relationship between intergenerational support and the older population subjective wellbeing, with economic support exerting the strongest effect, followed by emotional and instrumental support. Moreover, self-efficacy plays a partial mediating role in this relationship, while community environment and filial expectations have significant moderating effects. These results reveal that the formation of subjective wellbeing among the older population is a dynamic process shaped by multidimensional interactions across family, psychological, social, and cultural levels.

Further heterogeneity analysis reveals that female and rural the older population are more sensitive to emotional support, whereas male and urban the older population rely more on economic and instrumental support. This disparity reflects the profound impact of gender division of roles and urban–rural development imbalances on the sources of the older population wellbeing. It also suggests that policymakers should fully consider group differences and diverse needs when designing support systems for an aging society.

### 7.2 Policy implications

Strengthen family support functions and improve intergenerational assistance mechanisms. Research indicates that the family remains the most direct and emotionally meaningful source of support for enhancing the older population wellbeing. Governments should encourage and support children’s involvement in providing economic, daily, and emotional support to their older population parents through measures such as tax incentives, caregiving allowances, and family care subsidies. Additionally, diversified care models—such as intergenerational co-residence and neighborhood mutual assistance—can be explored to establish a support system characterized by “family-centered, community-assisted” care.

Improve community environments to amplify the effects of family support. A high-quality community environment can significantly enhance the impact of intergenerational support. Investments should be increased in public service facilities, age-friendly infrastructure, psychological support services, and spaces for social participation. Special attention should be given to enriching the spiritual and cultural life of the older population and facilitating social interactions, thereby enabling communities to play a positive moderating role in shaping the wellbeing of the older population.

Emphasize cultural guidance to activate the positive social function of filial piety. The moderating role of filial expectations indicates that traditional filial piety continues to exert a profound influence on the older population psychological perceptions and family relationships. Public campaigns, community education, and policy advocacy should integrate the spirit of filial piety with modern family responsibilities, fostering a societal culture of “respecting, honoring, and caring for the older population.” This approach can enhance the psychological and emotional value of intergenerational support.

Enhance the older population psychological resilience and self-efficacy. Self-efficacy serves as a crucial bridge between intergenerational support and subjective wellbeing. Therefore, beyond material and emotional support at the family and community levels, psychological health services and empowerment interventions targeting the older population should be strengthened. For example, offering mental health education, interest-based clubs, and community volunteer programs can boost the older population confidence and sense of control in coping with life’s challenges.

### 7.3 Limitations

Although this study provides new empirical evidence on the relationship between intergenerational support and the older population subjective wellbeing, several limitations remain and warrant further investigation in future research:

Limited causal inference. This study relies on cross-sectional data, which reveals associations among variables but restricts the ability to infer causality. Moreover, the dynamic mechanisms proposed in this study, particularly the pathways involving self-efficacy and the moderating role of community environment, cannot be fully verified using cross-sectional data. Future research should employ multi-wave longitudinal datasets, such as subsequent waves of the CFPS, to establish the temporal sequence among intergenerational support, psychosocial mediators, and subjective wellbeing. Such designs would provide stronger evidence for causal pathways and allow researchers to examine how these relationships evolve over time.

Scope for improved measurement. Subjective self-reported indicators were used to assess wellbeing and intergenerational support, which may introduce response bias. Future studies could incorporate more objective or multidimensional measures to enhance measurement accuracy.

Cultural and regional heterogeneity requires deeper exploration. While this study considered gender and urban–rural differences, it did not fully account for regional cultural variations or differences in social institutions. Future research could compare the effects of intergenerational support across provinces and cultural contexts to reveal heterogeneous mechanisms.

Insufficient consideration of public policy factors. The relationship between intergenerational support and wellbeing is not only a family issue but is also closely linked to social security systems, pension policies, and other public policies. Incorporating policy-level factors into future models could clarify how macro-level institutions shape micro-level wellbeing outcomes.

Overall, intergenerational support remains a key driver of the older population wellbeing, with self-efficacy, community environment, and filial expectations playing important mediating and moderating roles. In the context of rapid population aging, achieving the ideal of “well-cared-for, socially supported, and content in later life” requires coordinated efforts across family, community, and public policy domains. This not only reflects the continuity of family ethics and cultural values but also signifies progress in modern public governance and social civilization.

## Data Availability

The datasets presented in this study can be found in online repositories. The names of the repository/repositories and accession number(s) can be found below: https://www.isss.pku.edu.cn/cfps/sjzx/gksj/index.htm.
